# Molecular characterisation and control of *Acinetobacter baumannii* isolates resistant to multi-drugs emerging in inter-intensive care units

**DOI:** 10.1186/s12941-014-0036-2

**Published:** 2014-07-22

**Authors:** Ayşe Ertürk, Ayşegül Çopur Çiçek, Aziz Gümüş, Erkan Cüre, Ahmet Şen, Aysel Kurt, Alper Karagöz, Nebahat Aydoğan, Cemal Sandallı, Rıza Durmaz

**Affiliations:** Department of Infectious Diseases, School of Medicine, Recep Tayyip Erdogan University, Rize, Turkey; Department of Medical Microbiology, Faculty of Medicine, Recep Tayyip Erdogan University, Rize, Turkey; Department of Pulmonary Medicine, Faculty of Medicine, Recep Tayyip Erdogan University, Rize, Turkey; Department of Internal Medicine, Faculty of Medicine, Recep Tayyip Erdogan University, Rize, Turkey; Department of Anesthesiology And Reanimation, Faculty of Medicine, Recep Tayyip Erdogan University, Rize, Turkey; Department of Thoracic Surgery, Faculty of Medicine, Recep Tayyip Erdogan University, Rize, Turkey; Department of Microbiology Reference Laboratories, Turkey Public Health Institute, Molecular Microbiology Research and Application Laboratory, Rize, Turkey; Department of Biology, Faculty of Arts & Sciences, Recep Tayyip Erdogan University, Rize, Turkey; Department of Clinical Microbiology, Medical Faculty, Yildirim Beyazit University, Ankara, Turkey

**Keywords:** *Acinetobacter baumannii*, OXA, PFGE, Clonal relation

## Abstract

**Background:**

A nosocomial outbreak of *Acinetobacter baumannii* (AB) infections occurred among intensive care units (ICU) (surgery, medical, cardiovascular surgery, coronary unit) of Recep Tayyip Erdogan University Medical School (Rize, Turkey) between January 2011 and May 2012. The identification of isolates and clonal relation among them were investigated by molecular techniques.

**Methods:**

A total of 109 *AB* isolates were obtained from 64 clinical materials from 54 ICU patients and 3 from the hands of healthcare workers (HCWs) of 42 environmental samples. The isolates were identified by 16S rDNA sequencing and OXA- specific PCR. The clonal relation between isolates was investigated by PFGE methods using *Apa*I restriction enzyme.

**Results:**

All isolates were determined as *AB* by 16S rDNA sequencing and OXA-spesific PCR. While the *bla*_OXA-51-like_ gene was amplified in all isolates, the *bla*_OXA-23-like_ gene was amplified from 103 isolates. The PFGE pattern generated 9 pulsotypes and showed that the isolates from patients, HCWs, and the environment were genetically related. In 7 of these pulsotypes, there were 107 strains (98%) showing similar PFGE profiles that cannot be distinguished from each other, ranging from 2 to 53. The remaining 2 pulsotypes were comprised of strains closely associated with the main cluster. Two major groups were discovered with similarity coefficient of 85% and above. The first group consisted of 97 strains that are similar to each other at 92.7% rate, and the second group consisted of 12 strains that are 100% identical.

**Conclusions:**

The common utilization of the blood gas device among ICU was the reason for the contamination. *AB* strains can remain stable for a long period of time, although due to the disinfection procedures applied in hospitals, there is a small chance that the same clone might reappear and cause another epidemic. For that reason, the resistance profiles of the strains must be continuously followed with amplification-based methods, and these methods should be used to support the PFGE method in the short term.

## Background

*Acinetobacter baumannii (AB),* a non-fermentative, gram-negative bacteria, is an opportunist agent commonly existing in nature [1,2]. It can be alive for days on a lifeless surface and cause nosocomial infections in hospital environments [3-5]. *Acinetobacter* spp may especially cause ventilator-related pneumoniae and bacteriemia, urinary tract infections, secondary meningitis, and skin and wound infections [6,7]. *AB* strains that are frequently isolated from nosocomial infections are resistant to many agents including carbapenems [8-10]. Recently, *AB* epidemics have been reported that are resistant to all antibiotics except polymixines, and this situation has led to a search for new drug options [10-12]. The problems in the production of any antibiotic drug and the resistant nature of the bacteria have caused a need for preventive interventions against the infections. Recently, several molecular-based studies have shown that the specific clones resistant to carbapenem have been persistent, and there is a possibility of clonal expansion throughout the world [13-17].

This study assessed the epidemic level of *AB* found in intensive care units (ICU) (surgery, medical, cardiovascular surgery, coronary care unit) from Recep Tayyip Erdogan University Hospital (RTEUH) between January 2011 and May 2012. It also evaluated its source, probable avenues of contamination, and its clonal relations to determine the effective control measurements for *AB* in RTEUH, which is a public hospital with 450 beds. Located in the east Black Sea region of Turkey, the hospital services around half a million people. There are 9 beds in the surgery intensive care unit (SICU); 8 beds in the medical intensive care unit (MICU); 6 beds in the cardiovascular surgery intensive care unit (CVSICU), in which all of them have ventilator equipment; and 16 beds in the coronary care unit (CICU), in which two of them have isolation rooms with ventilatory equipment. In a one and a half year period (January 2011 through May 2012), the number of patients hospitalized for RTEUH was 51,828, of which 1,132 were admitted to intensive care units.

## Methods

### Bacterial isolates

One hundred and nine *AB* strains were collected from RTEUH between January 2011 and May 2012. Samples were obtained from each ICU separately, from intravenous (IV) catheters, medication solutions, IV solutions, ICU environments, surfaces of the study areas, ventilators, laryngoscopy knives, incubators, taps, sinks, drug containers, IV drop supporters, monitors, tables, feeding pumps, patients’ charts or fields, mechanic ventilation (MV) equipment, intubation tubes, intubation knives, resuscitation equipment, resuscitation cars, and blood gas devices located in the corridor, comprising 8 square meters between surgery and the CVSICU. At the same time, samples were collected from ICU workers’ hands by a direct cultivation process. These hand samples of health workers were collected immediately after direct contact with a patient who required an emergency procedure.

### Evaluating the epidemic

Based on data obtained by Hospital Infection Control Committee Team reports in January 2012, it was discovered that there was an *AB* epidemic in all ICUs during the last three months of year and simultaneously the preventive intervention had been started to get under control the epidemic. During the investigation, all suspect health procedures and infection control applications were analyzed. For the epidemiologic side of the investigation, cultures of the environment, equipment, hands of health workers, and other aforementioned items were evaluated. Because of the probability that the same strain caused the epidemic in the other four ICUs, the focus was directed to common contamination sources, common areas, equipment, transfer conditions, and personnel factors*.* The *AB* epidemic was not previously observed in the CVSICU previously and it was caused in the operating room. The blood gas instrument, frequently used during the cardiovascular surgery and commonly used temporarily by the SICU, CVSICU, CICU, and MICU, was examined to reach the source of contamination. At the beginning of May, a second investigation began because the cases continued to appear. From mid-January onward, on the first day a patient was admitted to any ICU and then weekly thereafter, cultures of the pharynx, axilla, and rectum of each patient were scanned.

### Identification of *A. baumannii* isolates

The samples obtained were cultured on bloody agar and EMB agar, and conventional methods plus a VITEK-2 Compact Automated System (bioMerieux, Marcy-l’Etoile, France) were used for bacterial identification and antibiograms. The antibiotic susceptibility of the isolated strains was determined by the Kirby Bauer disk diffusion method (CLSI 2010) [18]. Ampicillin-sulbactam (10/10 μg), piperaciline-tazobactam 100/10 μg, cefepime (30 μg), ceftazidime (30 μg), imipenem (10 μg), meropenem (10 μg), gentamicin (10 μg), amikacin (30 μg), tobramicin (10 μg), netilmicin (30 μg), trimethoprim/sulfamethoxazole (1.25 μg/23 μg), ciprofloxacin (5 μg), colistin (10 μg), and tigecycline (15 μg) antibiotic disks (Oxoid, UK) were used. *P.aeruginosa* ATCC 27853 was studied as the quality control strain. For colistin, the interpretive criteria of Galani et al. [19], and for tigecycline, the interpretive criteria of Jones et al. [20], were applied. The imipenem-resistant strains were detected by the disk diffusion method and confirmed by the imipenem E-test (Oxoid M.I.C Evaluator, UK).

### 16S rRNA gene sequencing of *A. baumannii*

Molecular identification of the isolates was performed by a 16S rRNA gene sequencing. *AB* isolates were grown in Luria Broth at 37°C overnight and collected by centrifuge at 13,000 rpm for 5 min. A pellet was suspended in 300 μl of distilled water and boiled for 10 min. It was then centrifuged again at 13,000 rpm for 20 min. The supernatant was transferred into a new tube and used as a DNA template. The oligonucleotide primers of 27 F (5′-AGAGTTTGATCMTGGCTCAG-3 as forward) and 1492R (5′-GGYTACCTTGTTACGACTT-3′ as reverse) (Macrogen) were used to amplify the 16S rRNA gene for isolate. The polymerase chain reaction (PCR) contained 10 μl of 5X *Taq* DNA polymerase reaction buffer (Promega), 200 mM of each dNTP, 10 pmol of the opposing primers, 1.5 U of GoTaqFlexi Polymerase (Promega, USA), 3 mM of MgClμ, and 5 μl genomic DNA in a final volume of 50 μl reaction volume. The PCR was performed under the following conditions: 2 min initial denaturation at 94°C; 35 cycles of denaturation (45 s at 94°C), annealing (60 s at 55°C), and extension (60 s at 72°C); and a final extension at 72°C for 10 min. Finally, the PCR product was analyzed by electrophoresis in 1% agarose gel and then visualized under UV light by staining with ethidium bromide. The PCR product was sent to Macrogen (The Netherlands) for sequencing. The same primer pairs were used for sequencing. The obtained sequence was analyzed by BLAST searches using the NCBI GenBank database [21].

### Multiplex PCR for detection of *bla*_OXA_ genes

Multiplex PCR was used for detecting *bla*_OXA-51_-like, *bla*_OXA-23_-like, *bla*_OXA-40_-like, and *bla*_OXA-58_-like genes [22]; and *bla*_OXA-48_ [23] was investigated separately. The primers used in obtaining the PCR amplifications and the expected size of those PCR amplifications are shown in Table 1. DNA extracts were conducted using a boiling method. PCRs were performed in a final volume of 50 μL and included 5 μL of genomic DNA, 20 pM of each primer, 10 μL reaction buffer (Promega), 3 μL 25 mM MgCl2, 200 μM of each dNTPs, and 1.5 U of *Taq* Polymerase (Promega). PCR amplification conditions were as follows: initial denaturation at 94°C for 3 min followed by 30 cycles of 25 s at 94°C, 40 s at 52°C and 50 s at 72°C, with a final extension of 5 min at 72°C. All PCR results were analyzed on 1% agarose containing 0.5 mg/L ethidium bromide and subsequently visualized under UV light.

**Table 1:** **The primer pairs used for amplification of OXA genes**

**Primer**	**5′→3′**	**Amplicon size**	**References**
OXA-51	F: TAATGCTTTGATCGGCCTTG	353	22
	R: TGGATTGCACTTCATCTTGG		
OXA-23	F: GATCGGATTGGAGAACCAGA	501	22
	R: ATTTCTGACCGCATTTCCAT		
OXA-24	F: GGTTAGTTGGCCCCCTTAAA	246	22
	R: AGTTGAGCGAAAAGGGGATT		
OXA-58	F: AAGTATTGGGGCTTGTGCTG	599	22
	R: CCCCTCTGCGCTCTACATAC		
OXA-48	F:TTGGTGGCATCGATTATCGG	733	23
	R:GAGCACTTCTTTTGTGATGGC		

### Pulsed-field gel electrophoresis (PFGE) analysis

The clonal relation among *AB* strains were analyzed according to the suggested protocol, by Durmaz et al. [24], using the *Apa*I restriction enzyme for the PFGE method. After PFGE, a dendogram analysis of band profiles was carried out using the Bionumerics (Applied Maths, Inc., Belgium, 6.01 version) program. In the evaluation of the clonal relations, the criteria suggested by Tenover et al. was used [25].

## Results

**Figure 1:**
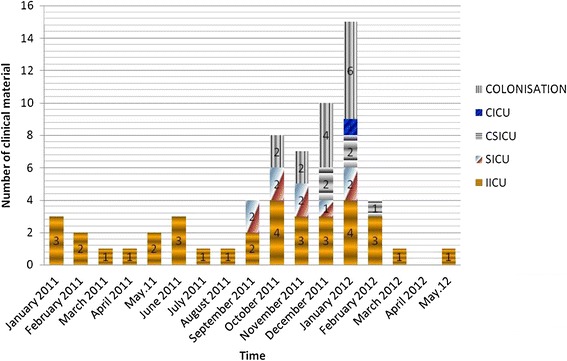
**The distribution of all** ***A.baumannii*** **isolates obtained from in all intensive care units according to months.**

One hundred and nine *AB* isolates were collected from 64 clinical materials (3 of bronchoalveloar lavage, 2 of urine, 8 of blood, 2 of catheter, 1 of gaita, 1 of vagina, 4 of wound, 43 of tracheal aspirate cultures) from a total of 54 patients, 42 samples from a total of 233 environmental samples, and 3 from workers’ hands from a total of 18 personnel. Fifty *AB* isolates, obtained from 64 clinical samples from all four ICUs, were the cause of the hospital infection (35 of them were ventilator-related pneumoniae, 10 of them were bacteremia, 2 of them were urinary tract infections, and 3 of them were surgical area infections), and the rest (14) were due to colonization (Figure 1). All isolates were confirmed by biochemical characterization and 16S rDNA sequencing analysis. A hospital infection diagnosis was carried out using Centers for Disease Control and Prevention (CDC) criteria. Infection speed and invasive tool use rates were calculated in accordance with the requirements of the National Hospital Infections Surveillance Web (NHISW). Nineteen of the 54 patients infected with *AB* died.

**Figure 2:**
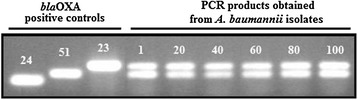
***bla***_**OXA**_ **gene profils of** ***A.baumannii*** **strains obtained from all clinical and environmental samples.**

**Figure 3:**
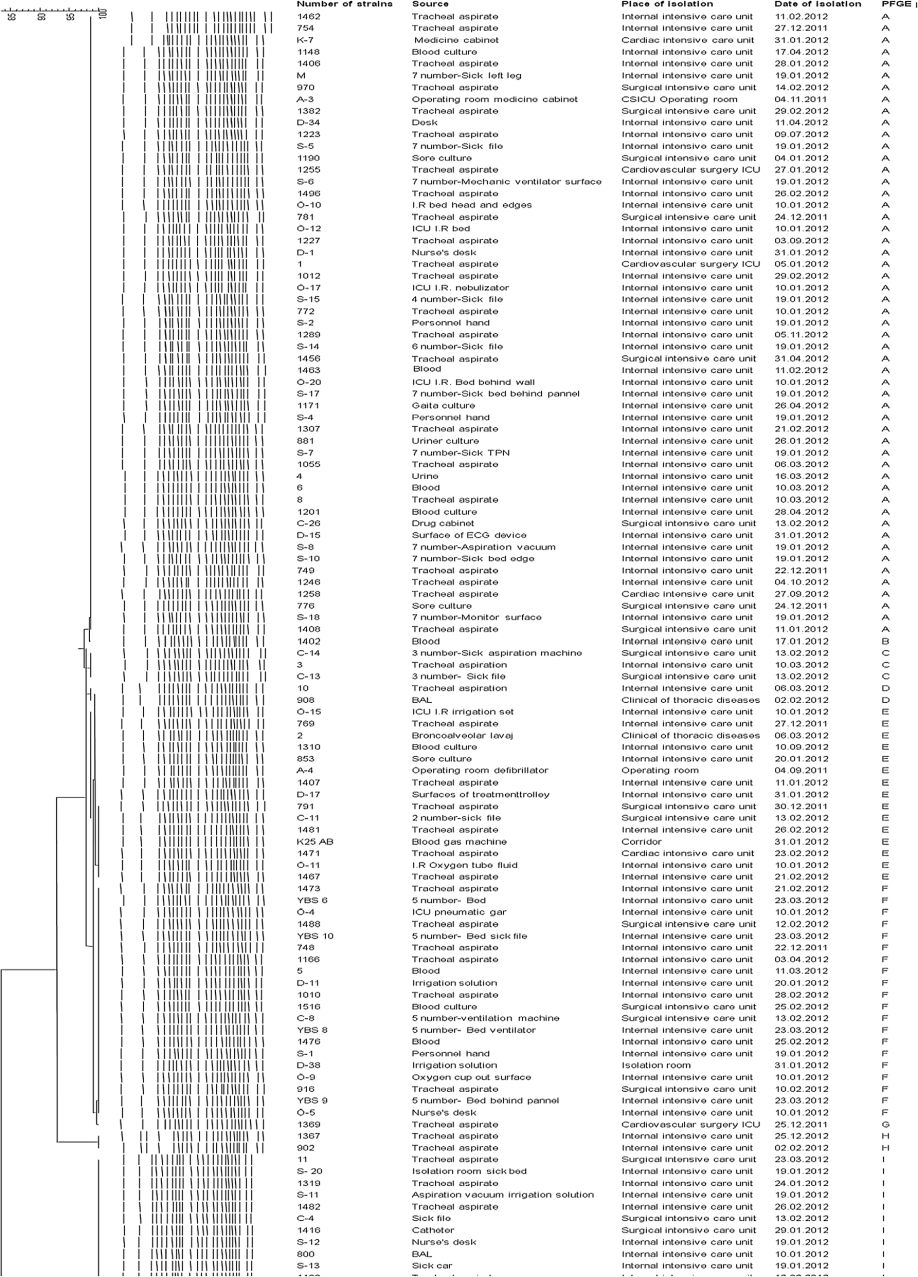
**The PFGE profiles of the 109** ***A. baumannii*** **outbreak isolates.**

All isolates were resistant to carbapenems and multiple antibiotics but sensitive to colistin and tigecycline. The highest resistance rate was found for ciprofloxacin, followed by piperacillin-tazobactam, with a 97.2% rate, and cefepime (Table 2). Imipenem and meropenem resistance were found at 91.9% and 93.5%, respectively. An E-test was carried out on all imipenem resistant strains; all were found as ≥ 32 μg/mL. *bla*_*OXA-51-like*_ was found as positive in all 109 strains, whereas *bla*_OXA-23-like_ was positive in 103 strains, while *bla*_OXA-24-like_, *bla*_OXA-48-like_, and *bla*_OXA-58-like_ were not determined (Figure 2). Nine pulsotypes were detected by evaluating all 109 strains with PFGE, using an *Apa*I restriction enzyme. In 7 of these pulsotypes, there were 107 strains (98%) showing similar PFGE profiles that could not be distinguished from each other, ranging from 2 to 53. Two of the pulsotypes were comprised of a strain closely associated with the main cluster. Given the similarity coefficient as 85% and above, two major groups were discovered. The first group consisted of 97 strains that are similar to each other at a 92.7% rate, and the second group consisted of 12 strains that are 100% identical (Figure 3).

**Table 2:** **The antibiotic sensitivities of** ***A. baumannii*** **isolates and screening results of OXA genes**

	**SAM**	**TZP**	**CAZ**	**FEM**	**AMK**	**GAT**	**TOB**	**NET**	**CIPX**	**TGC**	**CST**	**SXT**	**IPM**	**MEM**
Sensitive n (%)	8 (7.3)	3 (2.8)	19 (17.4)	2 (1.7)	19 (17.4)	85 (77.9)	86 (78.9)	93 (85.3)	2 (1.8)	109 (100)	109 (100)	69 (63.3)	7 (6.4)	6 (5.5)
Intermediate n (%)	22 (20.2)	0	7 (6.4)	1 (1.1)	4 (3.7)	3 (2.8)	0	1 (1.0)	0	0	0	0	2 (1.7)	1 (1.0)
Resistant n (%)	79 (72.5)	106 (97.2)	83 (76.2)	106 (97.2)	86 (78.9)	21 (19.3)	23 (21.1)	15 (13.7)	107 (98.2)	0	0	40 (36.7)	100 (91.9)	102 (93.5)
*bla*_OXA-51-like_	All isolates (n=109) were positive													
*bla*_OXA-23-like_	103 of isolates were positive													
*bla*_OXA-24-like_	All negative													
*bla*_OXA-48-like_	All negative													
*bla*_OXA-58-like_	All negative													

SAM: Ampicillin-sulbactam, TZP: Piperacillin-tazobactam, CAZ: Ceftazidime, FEM: Cefepime, AMK: Amikacin, GAT: Gentamicin, TOB: Tobramicin, NET: Netilmicin.

TGC: Tigecycline, CIPX(Ciprofloxacin),CST: Colistin, SXT: Trimethoprim-sulphamethoxazole, IPM: Imipenem, MEM: Meropenem.

During the epidemic, control strategies were determined with the assistance of an infectious disease specialist, an infection control nurse, and responsible doctors of all four ICUs and their nurses. It was separated blood gas device utilization as the first strategy and increased the number of these devices to four: one for surgery and the CVSICU, one for the MICU, one for the CICU, and one in emergency services. The devices were blocked for use and they were made available to health care workers when absolutely necessary. The second strategy was placing affected patients in isolated rooms. With very strict contact measures, all health personnel working in ICUs were informed one by one of the need for the prevention of cross-contamination when using their hands. The importance of controlling hand hygiene and environmental pollution were highlighted. Disposable gloves and gowns were provided for all personnel caring for affected patients, and permission to enter the isolated rooms was granted to only a few necessary individuals. The third strategy was the blocking of patient transfers between the ICUs and exchange of personnel. The fourth strategy limited environmental pollutions in the ICUs, as all ICUs were cleaned thoroughly. All surfaces were completely cleaned with 600 ppm sodium dichloroisocyanurate. Isolated rooms also were cleaned last and separately. The fifth strategy aimed to prevent contamination or pollution of the environment during endotracheal aspirations by using a closed aspiration system. A closed aspiration system was introduced to all patients who required mechanical ventilation; for those who did require this mechanical aspiration, aseptic open aspiration techniques were followed. The control measures were followed until two negative culture results during weekly interval checks were reported. After patient discharge (or after exitus) from isolated rooms, the rooms were completely disinfected. After confirmation of negative environmental culture results, new patients were hospitalized. Special agents were used, such as carbapenem, which require a unique, restrictive, computerized prescription system that can only be accessed with approval of an infectious disease specialist. During the epidemic, this antibiotic policy was maintained.

From January 2011 onward, the number of new diagnosed cases increased. Although environmental cleaning and preventive measurements were conducted every month, pre- and post-cleaning strains were persistent. In January 2012, the number of new cases peaked, and in the following two months, the new epidemic control measures began to take hold and the number of cases started to decrease. No units were closed. After May, only one new case, requiring colonization, was detected in a health care worker.

## Discussion

*AB* is an infectious species that causes severely progressive infections, and some of its isolates are resistant to most of antibiotics [25]. Although it is already resistant to multiple types of antibiotics, including aminoglycosides and fluoroquinolones, there has been an increase in recent years in the rates of carbapenem (potent antibiotic)-resistant *AB* strains. Carbapenem-hydrolyzing β-lactamases (carbapenemase) and molecular D class (OXA type enzymes) have been found responsible for the basic mechanism of resistance [26]. Hospital epidemics caused by carbapenem-resistant *AB* (CRAB) have caused concern throughout the world [27]. In recent years, *AB* epidemics due to carbapenem-resistant strains have increased in ICUs [28,29].

According to Ministry of Health 2012 National Hospital Infections Surveillance Web reports, antimicrobial resistance rates (percentiles) were as follows: throughout Turkey, in 315 hospitals with 170 units, the number of carbapenem-resistant *AB* was 9,196, the number of resistant strains was 7,114. The number of CRAB was 3145, the number of resistant strain was 2627. In our study, all strains were found to be sensitive to colistine and tigecycline. The biggest resistance was seen in ciprofloxacin with a 98.2% rate, followed by piperacillin-tazobactam and cefepime with a 97.2% rate. Imipenem resistance was 91.9% and meropenem resistance was 93.5%. It could well be attributable by the frequent use of empirical antibiotics such as cephalosporins and quinolones in icus

It could well be stemmed by the use of empirical antibiotics such as cephalosporins and quinolones in ICUs. Compared to the carbapenem resistance rate with other reported rates in our country, 50% to 75% of the rates were associated with a lower sample size. Previous studies have shown that each hospital, and even each nation, has to develop infectious control strategies against multi-drug resistant *AB* using a multidisciplinary approach [7,30,31]. These strategies, including the use of covered aspiration systems and appropriate antibiotic use, will control comprehensive environmental decontaminations [30-32]. Thus, several studies showed that some epidemics can be controlled by such measures [30-34], although extremely persistent strains may be very difficult to control. This situation requires continuing follow-up [35,36].

In the literature, total genom polymorphism with PFGE is the most reliable method for assessing the epidemiology of epidemics, and this is accepted as a “gold standard” for genotyping [24,30,37,38]. According to the results of this study, PFGE has enough discriminatory power for clonal separation in follow-up of both short- and long-term periods, but strains could well obtain the *bla*_OXA_ genes from different strains or microorganisms in time. Thus, it is imperative that the resistance profiles of strains must be continuously followed with amplification-based methods, and these methods should be used to support the PFGE method in the short term. According to the PFGE method, the one clone is responsible for *Acinetobacter* epidemics in hospitals.

Our results showed that the same strain has been responsible for contamination in all the ICUs, stemming from commonly used equipment that was contaminated by workers. An evaluation of contamination sources, using cultures from patients and environmental samples, has indicated that the epidemic might stem from commonly used blood gas analysis devices. Working personnel could well be responsible for the environmental contamination and the resulting cross-contamination. Seen retrospectively, the persistence over a period of months of the same strain in four different ICUs raised doubts about the contamination source. Infection control strategies, such as decreasing environmental pollution and using antibiotics appropriately, were employed while using equipment and transporting patients and in tactile contact with health care workers. Although there was *AB* persistency, a decrease in cases was achieved before reaching an epidemic level causing units to be shut down.

In conclusion, OXA type beta-lactamases have traveled swiftly among CRAB strains, consequently bringing carbapenem resistance to dangerous levels. These strains could be stable for a long time, although the disinfection procedures applied in hospitals could result in a slight chance that the same clone might cause another epidemic. In this context, to prevent persistent bacteria from causing hospital infections like *AB*, it is imperative that prevention and follow-up procedures be conducted on a continuous basis, and there is a need for more comprehensive molecular follow-up studies.
